# Changed Resting-State Brain Signal in Parkinson's Patients With Mild Depression

**DOI:** 10.3389/fneur.2020.00028

**Published:** 2020-01-31

**Authors:** Min Wang, Haiyan Liao, Qin Shen, Sainan Cai, Hongchun Zhang, Yijuan Xiang, Siyu Liu, Tianyu Wang, Yuheng Zi, Zhenni Mao, Changlian Tan

**Affiliations:** ^1^Department of Radiology, The Second Xiangya Hospital, Central South University, Changsha, China; ^2^Department of Radiology, The First Affiliated Hospital, University of Science and Technology of China, Changsha, China

**Keywords:** parkinson's diseases, mild depression, early treatment, regional homogeneity, resting-states, functional MRI

## Abstract

**Background:** Depression is reported to occur 5–10 years early than the onset of motor symptoms in Parkinson (PD) patients. However, markers for early diagnosis of PD in individuals with sub-clinical depression still remain to be identified.

**Purpose:** This study utilized Regional Homogeneity (ReHo) to investigate the alterations in resting state brain activities in Parkinson (PD) patients with different degrees of depression.

**Methods:** Twenty non-depressed PD patients, twenty mild to moderately depressed PD patients, and thirteen severely depressed PD patients were recruited. Hamilton Depression Scale (HDS) and the Beck Depression Inventory (BDI) were assessed depression. Resting-state functional magnetic resonance imaging (rs-MRI) was analyzed with ReHo.

**Results:** PD patients with mild to moderate depression had decreased ReHo in the left dorsal anterior cingulate cortex when compared with PD patients without depression. PD patients with severe depression exhibited increased ReHo in the left inferior prefrontal gyrus and right orbitofrontal area when compared with PD patients with mild to moderate depression. ReHo values in the bilateral supplementary motor area (SMA) in PD patients with severe depression was also increased when compared with PD patients without depression.

**Conclusions:** This study suggests that rs-MRI with ReHo analysis can detect early changes in brain function that associate with depression in PD patients, which could be biomarkers for early diagnosis and treatment of PD related depression.

## Introduction

Parkinson's disease (PD) is a common degenerative disease of the central nervous system with an incidence only secondary to Alzheimer's disease (1). Approximately 2% of people over 65 years old and 3% of people over 80 years old suffer from PD (2). It is predicted that the incidence and prevalence of PD will be twice as high as now by 2030 (3). Clinical symptoms of PD include motor symptoms and non-motor symptoms, but its treatments mainly focus on the motor symptoms. However, more and more studies found that non-motor symptoms often appeared several years or even decades before motor symptoms in PD patients (4), suggesting that these non-motor symptoms may be possible the signs for predicting PD occurrence. Among those non-motor symptoms, depression is the most common symptoms with a rate of 30–40% (5). A retrospective study in 32,415 Netherlanders demonstrated that depressive symptoms occurred 10 years in average before the motor symptoms in PD (6). A study in Americans revealed that depression was diagnosed about 5 years before motor symptoms in PD (7). In addition, tricyclic antidepressants can effectively treat PD-related depression, which can also subsequently delay the progression of PD disease (8). Reichmann's review proposed that it is not the depression to cause PD, but rather that a common pathology, such as the impairment and malfunction of dopaminergic, noradrenergic, and serotonergic systems, leads to depression, and motor symptoms sequentially (9). A previous study found that PD patients with mild depressive symptoms were six times more at risk to develop moderate to severe depressive symptoms than PD patients without depressive symptoms (10, 11). We therefore, hypothesize that a comparative study in PD patients with different degrees of depression may identify markers for the early detection of PD.

PD is thought to cause depletion of dopamine in the frontal and limbic systems. However, selective abnormalities in dopamine and other neurotransmitters in the limbic system were observed in PD patients with depression relative to PD patients without depression (12). Abnormalities in functional connectivity in the prefronto-limbic system (13) and structures in the prefrontal lobe, limbic system, and temporal lobe (14) were reported to be associated with depression in PD patients. Therefore, detecting the brain structural alterations in PD patients with depression may find markers for early diagnosis of PD. Functional magnetic resonance imaging (functional MRI, fMRI) is a non-invasive functional imaging technique that can detect changes in blood-oxygen-level-dependent (BOLD) signals. The fMRI includes a task state and a resting state for data acquisition and analysis. Resting-state functional MRI (R-fMRI) reflects the spontaneous neuronal activity. The data collected in the resting state is relatively stable, easy to obtain, and is therefore widely used in the study of brain function. Regional homogeneity (ReHo) refers to the similarity of BOLD signal changes of adjacent voxels in the same time series (15). The increase in ReHo indicates an increase in the consistency of neuronal activity in the local brain region (16). Previous studies with ReHo demonstrated that neural activity in the resting state is changed in multiple brain regions of PD patients (17–20). A study compared ReHo between PD patients with depression and PD patients without depression and found that PD patients with depression exhibited significant increases of ReHo in the left middle frontal gyrus and right inferior frontal gyrus, and decreases of ReHo in the left amygdala and bilateral lingual gyrus (21). Thus, a specific alteration in rs-MRI with ReHo analysis in PD patients with mild depression may be used as a marker for early diagnosis or prediction of PD.

This study investigated the alterations in resting state brain activities using ReHo in PD patients with different degrees of depression and non-depression.

## Materials and Methods

### Subjects

The patients with Parkinson's disease were recruited from the Department of Neurology, the Second Xiangya Hospital of Central South University from December 2015 to October 2018. Patients were diagnosed according to the British Brain Bank PD diagnostic criteria. PD patients were divided into PD without depression (ndPD, *n* = 20, 9 females), PD with mild to moderate depression (mdPD, *n* = 20, 8 females), and PD with severe depression (sdPD, *n* = 13, 7 females) according to the Hamilton Depression Scale (version 17) and the Beck Depression Inventory (BDI). In the ndPD group, the Hamilton score was <7 while BDI was <10; in the mdPD group, the Hamilton score was between 17 and 24 points while the BDI was between 10 and 25; and in the sdPD group, the Hamilton score was more than 24 while the BDI was more than 25. The demographic and clinical data of all participants are shown in Table 1.

**Table 1:** Demographics and clinical details (mean ± SD).

	**ndPD**	**mdPD**	**sdPD**	***P*** **value**		
				**mdPD vs.ndPD**	**sdPD vs.mdPD**	**sdPD vs.ndPD**
Gender(male/female)	11/9	12/8	6/7			
Age (year)	58.40 ± 6.96	60.55 ± 7.21	63.92 ± 10.57	0.403	0.246	0.060
Duration of disease(month)	28.35 ± 18.98	34.50 ± 33.07	29.00 ± 22.24	0.456	0.554	0.994
UPDRS	43.10 ± 4.94	42.42 ± 7.1	50.46 ± 8.40	0.889	0.002	0.004
H-Y	1.62 ± 0.64	1.62 ± 0.80	1.88 ± 0.68	1.000	0.317	0.317
HAMD	3.15 ± 1.69	18.70 ± 1.45	29.84 ± 3.46	< 0.001	< 0.001	< 0.001
BDI	6.40 ± 2.28	16.95 ± 5.09	33.00 ± 5.88	< 0.001	< 0.001	< 0.001
MMSE	27.30 ± 2.65	25.70 ± 3.93	22.15 ± 4.54	0.175	0.009	< 0.001

*PD, Parkinson's disease; ndPD, PD without depression; mdPD, PD with mild and moderate depression; sdPD, nPD with severe depression; UPDRS, Unified Parkinson's Disease Rating Scale; HAMD, the Hamilton Depression Scale; BDI, the Beck Depression Inventory; MMSE, Minimum Mental State Examination; H-Y, Hoehn & Yahr stage*.

This study was approved by the Medical Ethics Committee of the Second Xiangya Hospital, Central South University. All subjects enrolled in the study were voluntarily involved in the study after fully understanding the risks, research purposes, etc. The signed informed consents were obtained from the subjects or their guardians.

### Inclusion Criteria

Patients were included in the study if (1) they complied with the diagnosis of PD in the British Brain Bank PD diagnostic criteria; (2) were right handed; (3) had no dementia; and (4) their clinical data were available and they can finish MRI examination.

### Exclusion Criteria

Patients were excluded from the study if (1) they had unsuitable or contraindicated MRI examinations; (2) they had claustrophobia; (3) they felt discomfort during MRI examination, and cannot continue to complete the examination; (4) they had a history of long-term alcohol abuse or other histories of drug abuse; (5) they had a definite cause for depression, and (6) they were left handed.

Two experienced neurological physicians collected all subjects' age, gender, medical history, and course of the disease with exact consistency. They also recorded the patient's Hoehn-Yahr grading according to the nervous system. A two-way random model was then used to calculate the intraclass correlation coefficient of Hoehn-Yahr grading. It was 0.961 (*P* < 0.001), suggesting an absolute consistency between two physicians. All patients completed the Parkinson's disease rating scale (UPDRS), the BDI, the Hamilton Depression Scale, and the Mini-mental state examination (MMSE) survey.

### Scanning Parameters

The scanning session was performed at the Radiology Department of Second Xiangya Hospital, Central South University, on a SIEMENS 3.0 T scanner. A total of 580 T2- weighted EPI scans were acquired during the entire functional run (TR = 2,500 ms, TE = 25 ms, FA = 90°, FOV = 240 × 240 mm, 39 axial slices, slice thickness 3.50 mm, no gap). Two more dummy scans were acquired before each run to allow the fMRI signal to reach a steady state. Before the functional run, an anatomical volume consisting of T2-weighted MPRAGE scans with the high spatial resolution was also acquired.

### MRI and Data Analysis

RS-fMRI images were preprocessed using the toolboxes data processing assistant for resting-state functional MR imaging (DPARSF; http://www.restfmri.net/forum/DPARSF) through an RS-fMRI data analysis toolkit (REST1.8; http://www.restfmri.net) running on MATLAB R2010a (Math-works). The first ten volumes of the functional images were discarded.

The imaging data were preprocessed by slice timing, realignment, co-registration to individual structural T1 scan, segmentation into gray matter (GM), white matter (WM), and cerebrospinal fluid (CSF), as well as spatial normalization to Montreal Neurological Institute coordinates (MNI) space using the normalization parameters estimated in DARTEL, with a resampling voxel size of 3 mm × 3 mm × 3 mm. A temporal filter (0.01–0.08 Hz) was used to decrease the effect of low-frequency drifts and physiological high-frequency noise. The linear trends were then removed. Finally, the generated images were spatially smoothed with a 6 mm full-width at half- maximum (FWHM) Gaussian kernel after ReHo analysis.

ReHo analysis was performed in DPARSF software. ReHo maps of each subject were obtained on a voxel-wise basis by calculating Kendall's coefficient of concordance (KCC) between the time series of a given voxel and those of its nearest neighboring 26 voxels (15). The greater ReHo value of a given voxel means the higher degree of localized temporal synchronization within a neighboring cluster. Then, the voxel ReHo was divided by the average ReHo value of the entire brain in each subject for the purpose of standardization.

### Statistical Analysis

Statistical analyses were conducted with SPSS 20.0 statistical analysis software (SPSS Inc. Chicago, IL, USA). The significant threshold was set to *p* = 0.05. The one-way analysis of covariance (ANCOVA) was used to compare the differences between ReHo values and other demographic data among the three groups. The significant differences were set at *P* < 0.05 (with a combined threshold of *P* < 0.05 and a minimum cluster size of 26 voxels), corrected by the AlphaSim program in the REST Software. A two-sample post hoc *t*-test was performed between each pair of the three groups.

## Results

There were no significant differences in gender, age, and duration of disease between the three groups. Additionally, head motion caused no significant differences between those patients (*p* > 0.05). There were significant differences in Unified Parkinson's Disease Rating Scale and Minimum Mental State Examination between sdPD and mdPD groups as well as between sdPD and ndPD groups. There were significant differences in the Hamilton Depression scores and BDI scores between any two groups among the three groups (Table 1).

**Figure 1:**
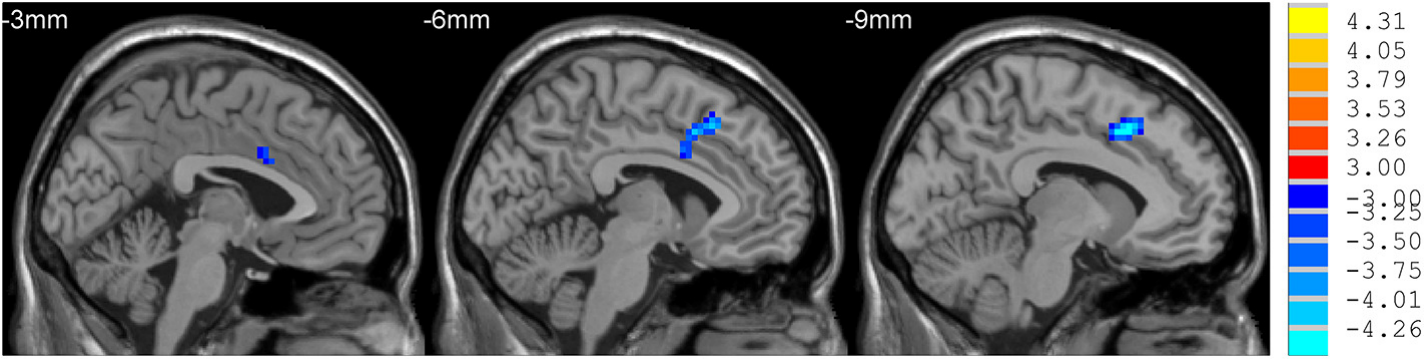
Comparison of ReHo between mdPD and ndPD group. There was a decrease in ReHo value in the left dorsal anterior cingulate cortex in PD patients with mild to moderate depression (*P* < 0.05).

**Figure 2:**
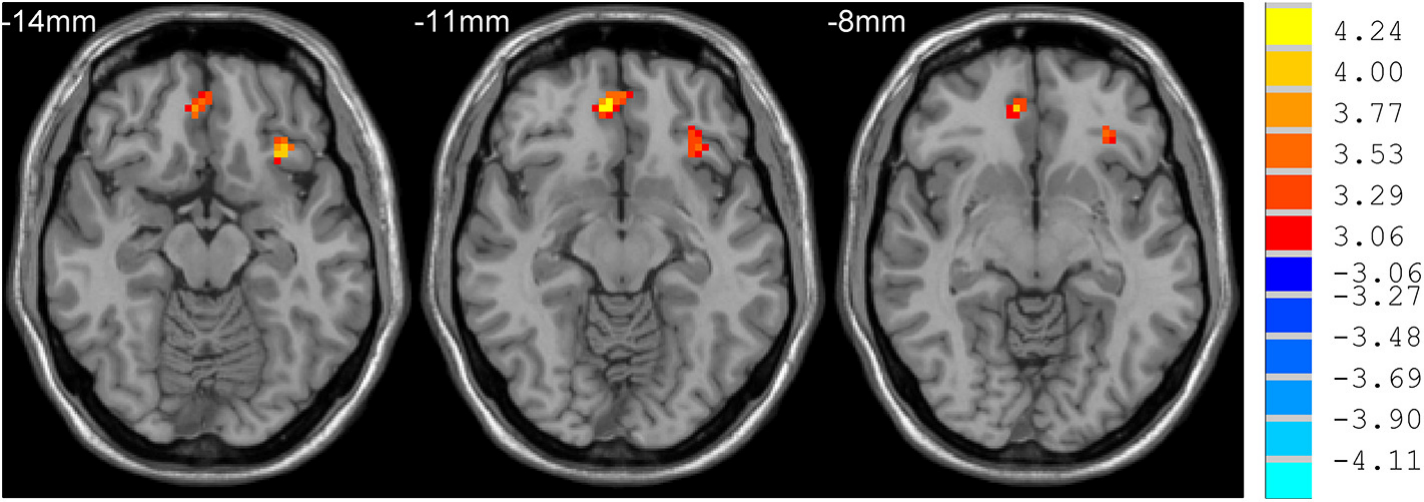
Comparison of ReHo between sdPD and mdPD group. There was an increase of ReHo value in the left inferior prefrontal gyrus and right orbitofrontal area in PD patients with severe depression (*P* < 0.05).

**Figure 3:**
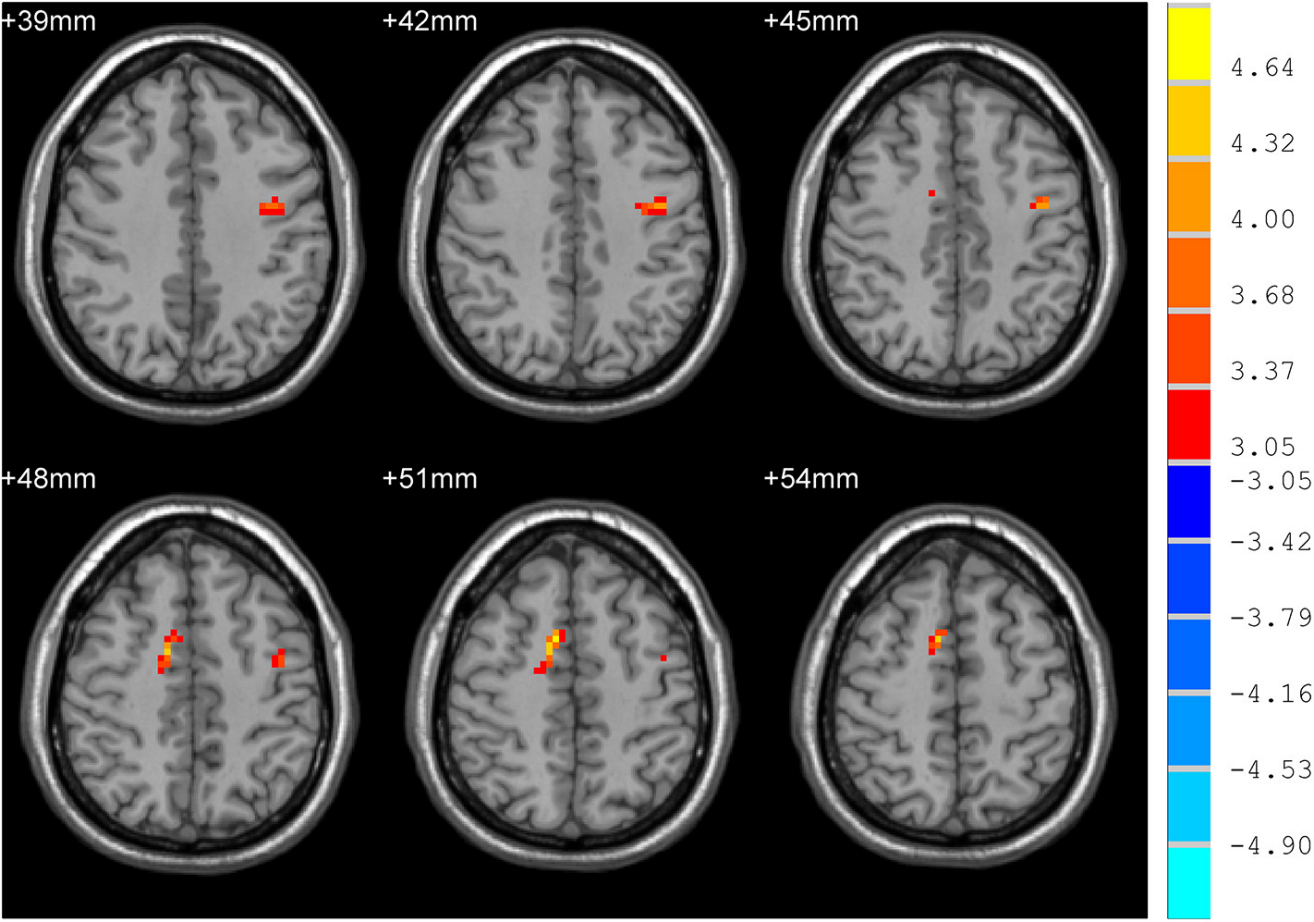
Comparison of ReHo between sdPD and ndPD group. There was an increase of ReHo values in the bilateral supplementary motor area in PD patients with severe depression (*P* < 0.05).

mdPD patients showed decreased ReHo values in the left dorsal anterior cingulate cortex when compared with ndPD patients (Figure 1). sdPD patients showed significant increased ReHo values in the inferior prefrontal gyrus and right orbitofrontal area when compared to mdPD patients (Figure 2). sdPD patients showed increased ReHo in the bilateral supplementary motor area (SMA) when compared with ndPD patients (Figure 3 and Table 2).

**Table 2:** Brain regions showing ReHo differences among groups.

**Brodmann Area**	**Brain regions**	**Cluster size (voxles)**	**Peak T values**	**Peak Coordinates in MNI**		
				**X**	**Y**	**Z**
**mdPD-ndPD**						
32	DPFC-L	41	−4.5075	−9	15	42
**sdPD-mdPD**						
47	IPG-L	39	4.1459	−33	27	15
11	OFA-R	28	4.4753	9	45	−12
**sdPD-ndPD**						
6	SMA-R	38	4.9573	9	6	51
6	SMA-L	30	4.2223	−39	−6	42

*PD, Parkinson's disease; ndPD, PD without depression; mdPD, PD with mild and moderate depression; sdPD, PD with severe depression; DPFC-L, left dorsolateral prefrontal cortex; IPG-L, left inferior prefrontal gyrus; OFA-R, right orbitofrontal area, SMA-R, right supplementary motor area, SMA-L, left supplementary motor area*.

## Discussions

Depression is thought not only a non-motor symptom of PD, but also a predictive marker for the occurrence of PD in its early stage. This study found that PD patients with mild to moderate depression had a reduced ReHo value in the dorsal anterior cingulate gyrus of the left cerebral hemisphere compared with PD patients without depression. Also, PD patients with severe depression had an increased ReHo value in the left inferior frontal gyrus (Brodmann 47) and right temporal gyrus (Brodmann 11) compared with PD patients with mild to moderate depression, as well as an increased ReHo value in the bilateral assisted motor zone (SMA) compared with PD patients without depression. Thus, this study suggests that rs-MRI with ReHo analysis may provide imaging marker for early diagnosis and prediction of PD in patients with sub-clinical depression.

The cingulate cortex is not only a vital hub in the default network, but also a critical hub in the limbic system. A previous study using resting-state fMRI and seed-based functional connectivity analysis revealed an increased functional connectivity between the ventral tegmental area and anterior cingulate cortex in depressed PD patients relative to healthy controls and non-depressed PD patients. Also, this aberrant connectivity correlated with the severity of depression in PD patients (16). Wang et al. study also observed degree centrality abnormalities in the anterior cingulate cortices in depressed PD patients compared to non-depressed PD patients (17). Skidmore et al. reported that the severity of depression in PD patients was positively correlated with the ALFF signal of the cingulate gyrus (22). In this study, we found a decreased ReHo value in the left dorsal anterior cingulate cortex of Parkinson patients with mild to moderate depression when compared with non-depressed PD patients. Thus, our study confirmed the involvement of the cingulate cortex in the depression of PD patients.

The prefrontal cortex includes the Brodmann Area 47 and 11. A meta-analysis revealed that repetitive transcranial magnetic stimulation over the prefrontal cortex exhibited a significant anti-depressive effect in PD patients (23). A SPECT study showed that the perfusion in the right medial orbitofrontal cortex was reduced in the depressed PD patients as compared with non-depressed PD patients (24). Sheng et al. study with ReHo and FC methods found that PD patients with depression had increased regional activity in the left frontal and medial frontal gyrus when compared with non-depressed PD patients. Brain network connectivity analysis demonstrated that the function of the prefrontal-limbic system was significantly weakened in depressed PD patients (21). Hu et al. study found that the connection of the left cingulate band back to the posterior cingulate gyrus, the anterior frontal lobes, the prefrontal lobes, and the inferior frontal gyrus was significantly enhanced in depressed PD patients compared to non-depressed PD patients (25). Our study also found that PD patients with severe depression had increased ReHo values in the left inferior prefrontal gyrus and right orbitofrontal area compared with patients with mild to moderate depression.

The supplementary motor area (SMA), the posterior third of the medial aspect of superior frontal gyrus, is involved in self-initiated motor movements, planning, and sequencing the motor action, response inhibition, and bimanual movements. A randomized, double-blind, sham-controlled, multi-center study revealed that the 1-Hz repetitive transcranial magnetic stimulation over the SMA was effective for improving the motor symptoms in PD (26). However, whether the SMA is associated with PD related depression remains to be further studied. In this study, we found that PD patients with severe depression had increased ReHo values in the bilateral SMA compared to PD without depression.

In our study, depressed PD patients had abnormal ReHo values both in the cingulate cortex and the orbitofrontal area. Luo et al. study with a resting-state functional MRI demonstrated that PD with depression showed significantly higher ALFF value in the left orbitofrontal area, which positively correlates with the scores of Hamilton Depression Rating Scale (27). However, Skidmore et al. study explored the relationships between depression and ALFF in patients with PD, and found that the severity of depression positively correlates with ALFF value in the right cingulate cortex (23). Our study suggests that it may also be associated with abnormal brain regions at different stages of depression in PD patients.

We acknowledge several limitations of the current study. First, the sample size in this study is relatively small and further studies from multiple centers with large sample size may provide more reliable conclusions. Second, although there was no statistical difference in the ages between three groups of PD patients, there was subtle difference. This may implicate that the effects of age were not completely ruled out. Third, this study used a combination of two scales to determine the degree of depression in Parkinson's patients. Patients with inconsistent grading of the two scales were not included in the study. This may have a bias.

In conclusion, PD patients with and without depression have different abnormal brain areas; PD patients with different degrees of depression have different abnormal brain regions; and rs-MRI with ReHo analysis may provide imaging evidence as markers for early diagnosis and prediction of PD in patients with sub-clinical depression.

## Data Availability Statement

The raw data supporting the conclusions of this article will be made available by the authors, without undue reservation, to any qualified researcher.

## Ethics Statement

The studies involving human participants were reviewed and approved by the Medical Ethics Committee of the Second Xiangya Hospital, Central South University. The patients/participants provided their written informed consent to participate in this study.

## Author Contributions

MW, HL, QS, YZ, and ZM: data collection. MW, HL, QS, SC, HZ, YX, SL, TW, YZ, and ZM: data collection and data analysis. MW and HL: manuscript writing. CT: project development and manuscript revising. All authors: read and approved the final manuscript.

### Conflict of Interest

The authors declare that the research was conducted in the absence of any commercial or financial relationships that could be construed as a potential conflict of interest.
